# Intraseasonal oscillation of deep currents influenced by mesoscale eddies in the Kuroshio Extension Region

**DOI:** 10.1038/s41598-019-39567-7

**Published:** 2019-03-11

**Authors:** Yansong Liu, Fei Yu, Feng Nan, Wenzheng Zhou

**Affiliations:** 1 Institute of Oceanology, Chinese Academy of Sciences, Qingdao, China; 2 College of Earth Science, University of Chinese Academy of Sciences, Beijing, China; 3 Qingdao National Laboratory for Marine Science and Technology, Qingdao, China; 4 CAS Key Laboratory of Ocean Circulation and Waves, Institute of Oceanology, Chinese Academy of Sciences, Qingdao, China; 5 Center for Ocean Mega-Science, Chinese Academy of Sciences, Qingdao, China

## Abstract

Intraseasonal oscillation of deep currents in the Kuroshio Extension region is examined using observations from a collection of current meter moorings. The moored observations reveal variability with characteristic time scales of 23–38 days for velocity time series and of 38–99 days for temperature time series. The time series of normalized temperature (NT) in the deep ocean change correspondingly with sea level anomaly (SLA). The maximum correlation coefficient between NT in the deep ocean and SLA is also up to 0.7. Positive correlation is observed between deep currents and surface geostrophic current. Furthermore, the influence of mesoscale eddies on deep currents is examined by analyzing the data collected when cyclonic and anticyclonic eddies crossed the current meter mooring. Whether anticyclonic or cyclonic eddy intensified the deep currents from 2000 m to 4000 m in the same direction and increased the amplitude. These results provide observational evidence of intraseasonal oscillation in the deep ocean and the effect of mesoscale eddies on deep currents in the Kuroshio Extension region.

## Introduction

The deep ocean influences global warming^1^ and the geochemical cycle of the Earth’s environment system because of its potentially huge capacity to transport and store heat and materials^2^. Since the last century, scientists have begun to study the deep ocean^3^ and investigate the pathway^4–6^ and strength^7,8^ of deep currents based on observations and model outputs. In Kuroshio Extension region, southward deep western boundary current (1–2 cm s^−1^) is along the slope inshore of the Japan Trench, and northward deep flow (4 cm s^−1^) is over the trench^6,9^. However, due to the lack of *in situ* observations from the deep ocean, especially long-term moored observations, the temporal variation of deep currents in the Kuroshio Extension region, including dynamic processes of intraseasonal oscillation, is lack of more being studied as the situation of deep currents.

The intraseasonal oscillation is a ubiquitous feature around the world ocean^10^. Intraseansonal variability of the surface currents have been extensively studied in the existing literature^11^. In comparison, intraseasonal oscillation of deep currents is much less explored, although such variability was reported in the western subpolar North Atlantic^12^, South China Sea^13^, western Pacific^14,15^ and near the East Pacific Rise^16^. In the Kuroshio Extension region, there are several experiments focused on the Kuroshio Extension jet, for example KESS (Kuroshio Extension System Study), KERE (Kuroshio Extension Regional Experiment) and “WESPAC” program. Variations of the deep currents on intraseasonal time scales have been detected by *in situ* measurements in Kuroshio Extension region. The kinetic energy spectra from current meter moorings as part of KESS showed that energy is largely confined to the 30–60 day band^17^. Energy in this band accounted for 25–50% of the total deep-pressure variance and was strongest^18^. However, the variation of deep current observed by moored current meter in the PCM-6 section of WOCE (World Ocean Circulation Experiment) is less analyzed.

Dynamic processes are keys to understanding temporal variation of the deep ocean. As the dominant mode of intraseasonal oscillations, the Madden-Julian Oscillation is defined from the tropical atmosphere. A response of the deep ocean to local wind forcing in a topographic Sverdrup balance at the intraseasonal time scales was demonstrated from analyzing a large number of deep current meter observations in the North Pacific Ocean^19,20^. The relationship between currents (as well as temperature) below the mixed layer and meteorological forcing at subinertial frequencies was also observed in the western North Atlantic^21^. Eastward-propagating oceanic equatorial Kelvin waves forced by Madden-Julian Oscillation were observed to extend downward to 1500 meters and have larger amplitude than the annual cycle using the Argo array of profiling floats^22^. In addition, the relative important of atmospheric forcing on bottom pressure intrasesonal variability was investigated by comparing *in situ* measurement with models^23,24^. Besides atmospheric forcing, the mesoscale eddies in the upper ocean also influenced multiscale flows in the deep ocean. The velocities at depth are associated with observed mesoscale eddies near the East Pacific Rise^16^, and in the South China Sea^25^ and Southern Ocean^26^. The numerical experiments^27,28^ was also used to simulate the correlation between intensified bottom current velocities and the passage of anticyclones. In the Kuroshio extension region, statistically significant coherence between 30–60 day upper- and deep-ocean streamfunction anomalies was demonstrated^18^. However, the relationship between mesoscale eddy and deep currents from *in situ* observation of Kuroshio extension region is also of interest but less reported.

The aim of present research is to provide an investigation for the intraseasonal oscillation of deep currents in the Kuroshio Extension region. Satellite observational data are analyzed to obtain characteristics of the upper ocean. The relationship between mesoscale eddy and deep currents is presented, and the underlying mechanism of that is also explored in this paper.

## Results

### Intraseasonal oscillation in the deep ocean

**Figure 1:**
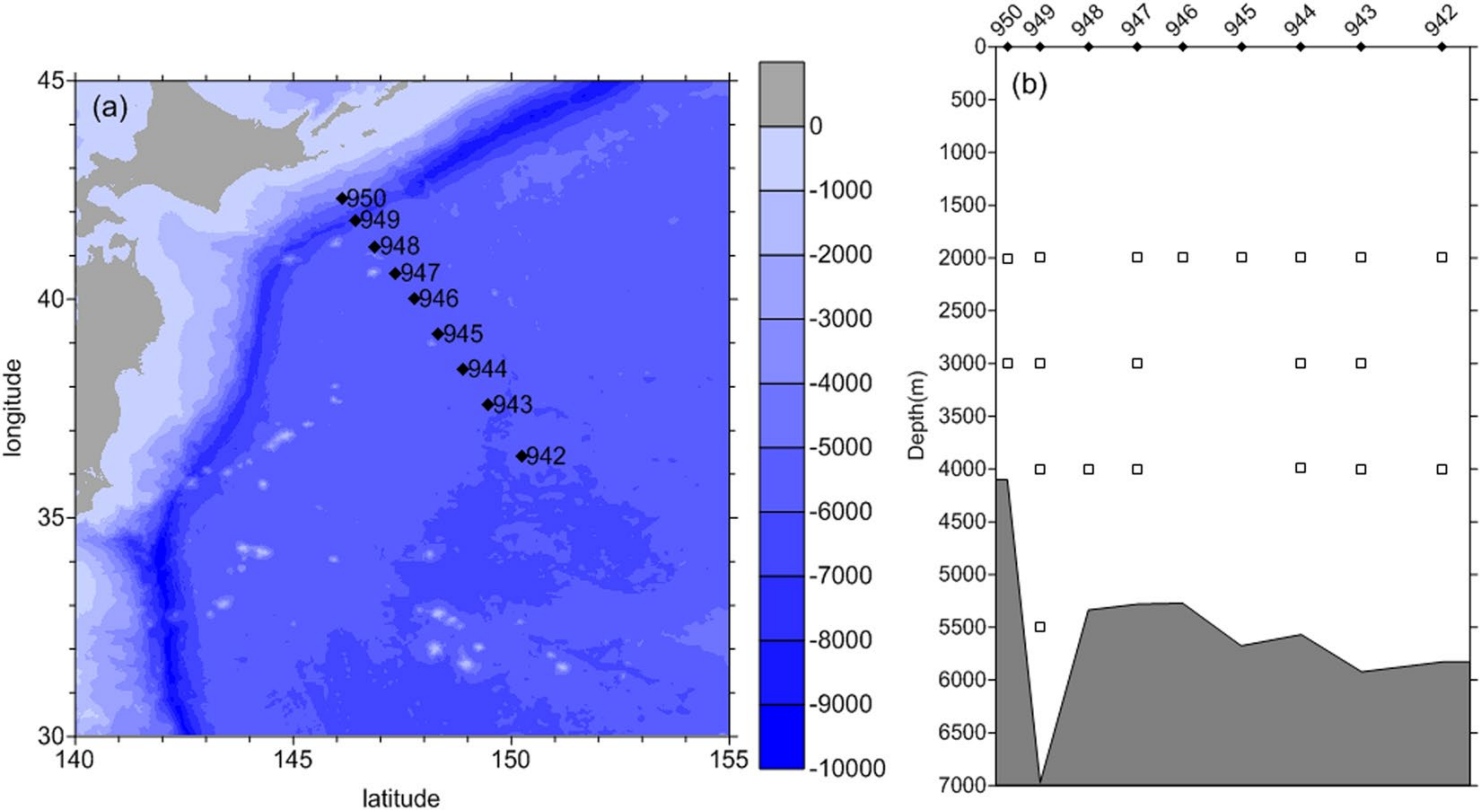
(**a**) Bathymetry of the Northwest Pacific from Etopo 1 dataset (shading) and the locations of current meters (black diamonds). (**b**) Vertical arrangement of current meters with coincident bathymetry. The open squares indicate depths with data record. Figures are plotted using MATLAB R2016a (http://www.mathworks.com/).

**Figure 2:**
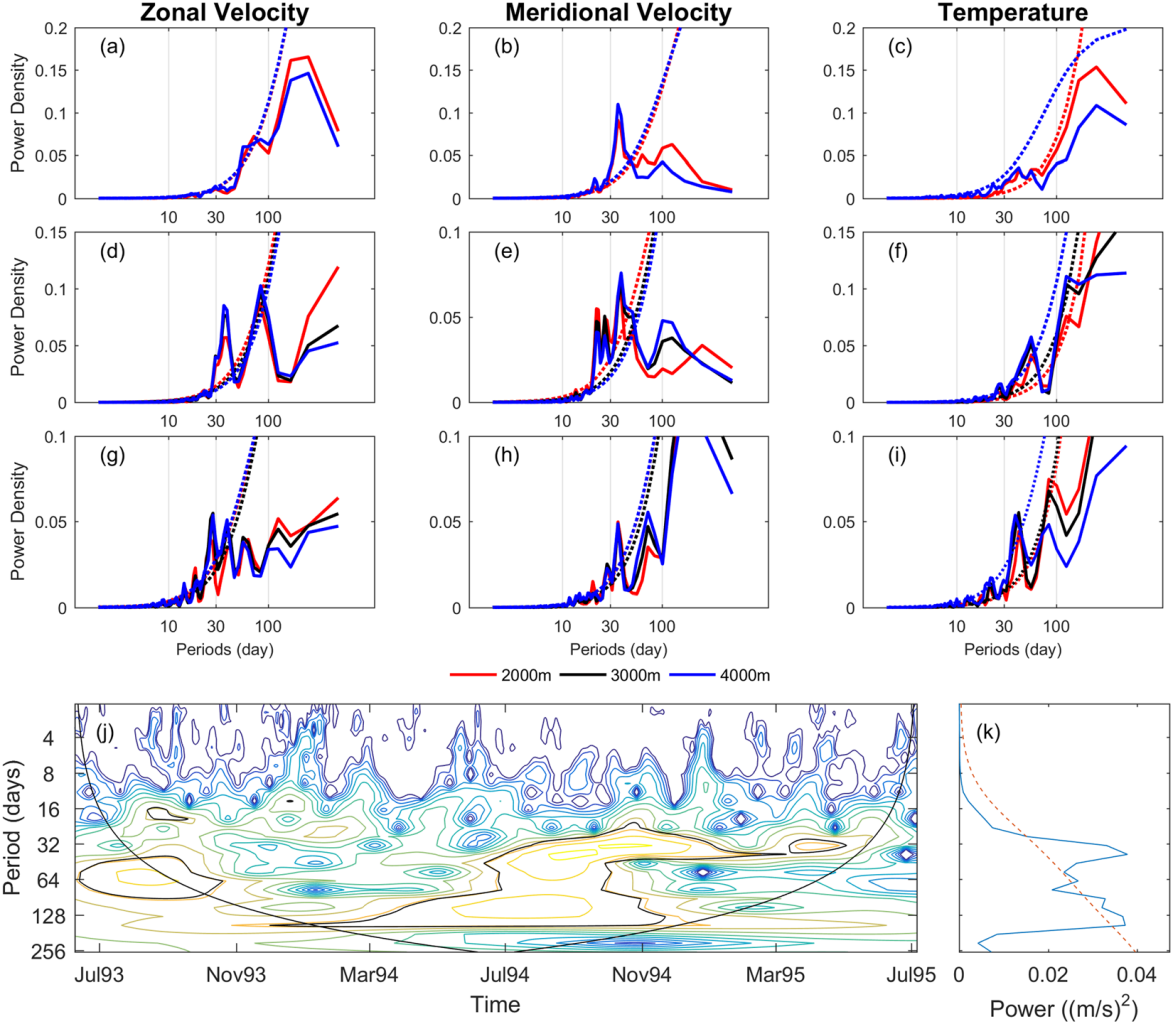
(**a–i**) Power spectra density (solid line) and 95% confidence level (dashed line) of zonal velocity (left column), meridional velocity (middle column) and temperature (right column) from CMs No. 942 (**a**–**c**), 944 (**d**–**f**) and 947 (**g–i**). The red, black, and blue line is the 2000, 3000, and 4000 m respectively. (**j**) The wavelet power spectrum of meridional velocity at CM No. 942, using the Morlet wavelet. The x-axis is the wavelet location in time, and the y-axis is the wavelet period in days. The black contours outline the significance regions and cone of influence, using a red-noise background spectrum. (**k**) Global wavelet spectrum (solid line) and significance level (dashed line). Figures are plotted using MATLAB R2016a (http://www.mathworks.com/).

As described by a previous study^6^, the section of PCM-6 was set along a line running from the continental slope off Hokkaido to the central Northeast Pacific Basin (Fig. 1a). Current meter (CM) No. 950 was at a water depth of about 4 km on the continental slope, 949 was in the center of the Kuril Trench, 948–944 on the Hokkaido Rise, and 943 and 942 at maximum water depths in mid-basin (Fig. 1b). The mean velocity was ~5 cm s^−1^ at CM No. 942, ~2 cm s^−1^ at CMs Nos. 943–946, and ~8 cm s^−1^ at CM No. 949. Besides, the depth of thermocline is around 500 m at CM No. 942, and sharply shallow to the north. Each mooring carried a CM at nominal depths of 2000, 3000, and 4000 m; and CM No. 949 in the trench had an extra depth at 5500 m (Fig. 1b). Because the time series were uninterrupted over the nominal two years, power spectral analysis of daily velocities (zonal and meridional) and temperature can be used to describe the temporal variability. The power spectra of CMs Nos. 942, 944 and 947 are shown in Fig. 2a–i. We only show these records as they represent different locations of the PCM6 section and have more records at different depths. Other results are shown in Supplementary Information. To check and verify the results of our spectral analysis, wavelet analysis is carried out for these time series. Figure 2j,k show the wavelet analysis of meridional velocity at 2000-m depth of CM No. 942. The result of spectral analysis shows a peak near 36 days (Fig. 2b); and the wavelet analysis and global wavelet spectrum (Fig. 2j,k) also shows a peak of 38 days. Another peak of wavelet analysis is 128 days, but it is slightly over the confidence curve. The results of spectral analysis will be used in this paper.

At CM No. 942, the spectra of meridional velocity have a peak near 36 days at both 2000 and 4000 m. The zonal velocity power spectra at CM No. 944 show a significant peak at 38 days, and the meridional velocity have a peak at 23–38 days. The velocity power spectra at CM No. 947 have similar peaks at 27–37 days, as CM No. 944. The temperature records reveal frequency peaks with 42, 54, and 42 days at CMs Nos. 942, 944, and 947, respectively. For the other moorings, both zonal and meridional velocity power spectra indicate 25–33 day peaks (Supplementary Fig. 1). The temperature data reveal peaks at 38–56 days. However, there are no obvious peaks in the power spectra of velocity at CMs No. 949 and No. 950. According to the vertical structure of the CMs from Fig. 1b, CM No. 949 was in the trench, and CM No. 950 was the closest to the shore. The topography may be one factor that influences the velocity. Although there are no significant peaks at CMs No. 949 and No. 950 in the velocity fields, both 45- and 99-days peaks can be seen in the temperature records. The other outstanding general feature of the power spectra is that the vertical difference at each site is very little. The peaks of power spectra show similar distributions through all depth levels from 2000 to 4000 m. This result is the same as previous research^6^ on mean velocity vectors. The deep current is highly barotropic below the depth of 2000 m, and has small vertical shear expect above the continental slope.

Previous observations of currents also showed energetic peaks of deep velocity with periods between 20 and 100 days^12–16^. The subinertial velocities at depth near the East Pacific Rise were significantly correlated with near-surface geostrophic currents estimated from sea-surface height data^16^. The observations in the South China Sea also evidenced the effect of observed mesoscale motions on benthic currents^13^. The location of the moorings used here is in the area where eddies frequently occur^29^. There exist intraseasonal oscillation in the upper ocean of the central North Pacific^30^, and the variance-preserving spectra of spatially smoothed SLA revealed a band from 20 days to 1 year. To examine the variability of upper-ocean fields, we examined the temporal characteristics of SLA in the Kuroshio Extension region.

**Figure 3:**
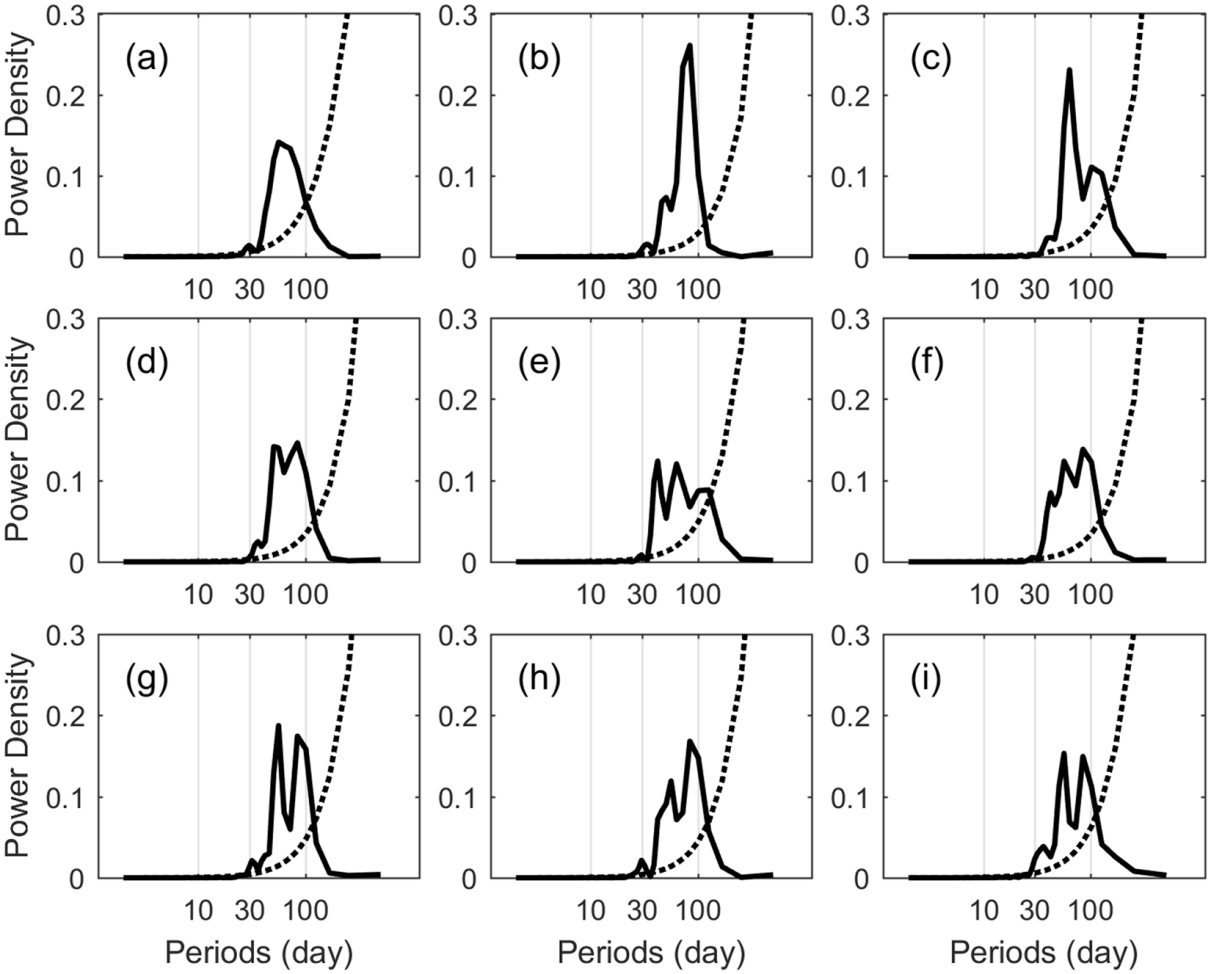
The power density (solid line) and 95% confidence level (dashed line) of filtered SLA at the closet site nearby CMs No. 942 (**a**) to No. 950 (**i**). Figures are plotted using MATLAB R2016a (http://www.mathworks.com/).

Figure 3 shows the power spectra of SLA at the sites near the CMs (the locations of the sites are given in Table 1) from June 8, 1993 to July 7, 1995. To focus on the intraseasonal time scale, the daily SLA data were high-pass filtered with a cut-off frequency of 100 days before power spectral analysis. The result reveals significant peaks centered near the 41–91 day period. Interestingly, the peak in the SLA has a period similar to the deep temperature. Especially for CMs No. 949 and No. 950, although they had no obvious peaks in the power spectra of velocity, their temperatures had peaks of 45 and 99 days, which corresponded with the peaks in SLA. The temporal variability of both SLA and deep-ocean temperature all show the intraseasonal signals. That means some connection existed between the upper- ocean and deep ocean. As we know, the negative and positive SLAs represent cyclonic and anticyclonic eddies, respectively. The relationship between mesoscale eddies in the upper ocean and currents in the deep ocean is presented next.

**Table 1:** Current meter information.

CM No.	CM Position		CM depth (m)	Record length (days)	Site of SLA		T_mean_	T_STD_
	Lon. (°N)	Lat. (°E)			Lon. (°N)	Lat. (°E)	(°C)	(°C)
942	36.4025	150.2307	2000	758	36.5	150.25	1.90	0.05
			4000	758			1.46	0.01
943	37.5858	149.4708	2000	756	37.5	149.5	1.94	0.05
			3000	756			1.57	0.02
			4000	756			1.48	0.01
944	38.4003	148.8978	2000	754	38.5	149	1.97	0.04
			3000	754			1.58	0.04
			4000	754			1.47	0.01
945	39.2022	148.3275	2000	752	39.25	148.25	1.95	0.03
946	40.0043	147.7727	2000	750	40.0	147.75	1.88	0.03
947	40.6	147.3492	2000	748	40.5	147.25	1.87	0.04
			3000	748			1.53	0.02
			4000	748			1.46	0.01
948	41.2083	146.8778	4000	746	41.25	146.75	1.46	0.01
949	41.8143	146.4203	2000	745	41.75	146.5	1.96	0.06
			3000	379			1.59	0.02
			4000	745			1.50	0.01
			5500	745			1.57	0.01
950	42.2853	146.1092	2000	744	42.25	146.0	1.92	0.04
			3000	744			1.54	0.02

### Relationship between upper- ocean and deep ocean

**Figure 4:**
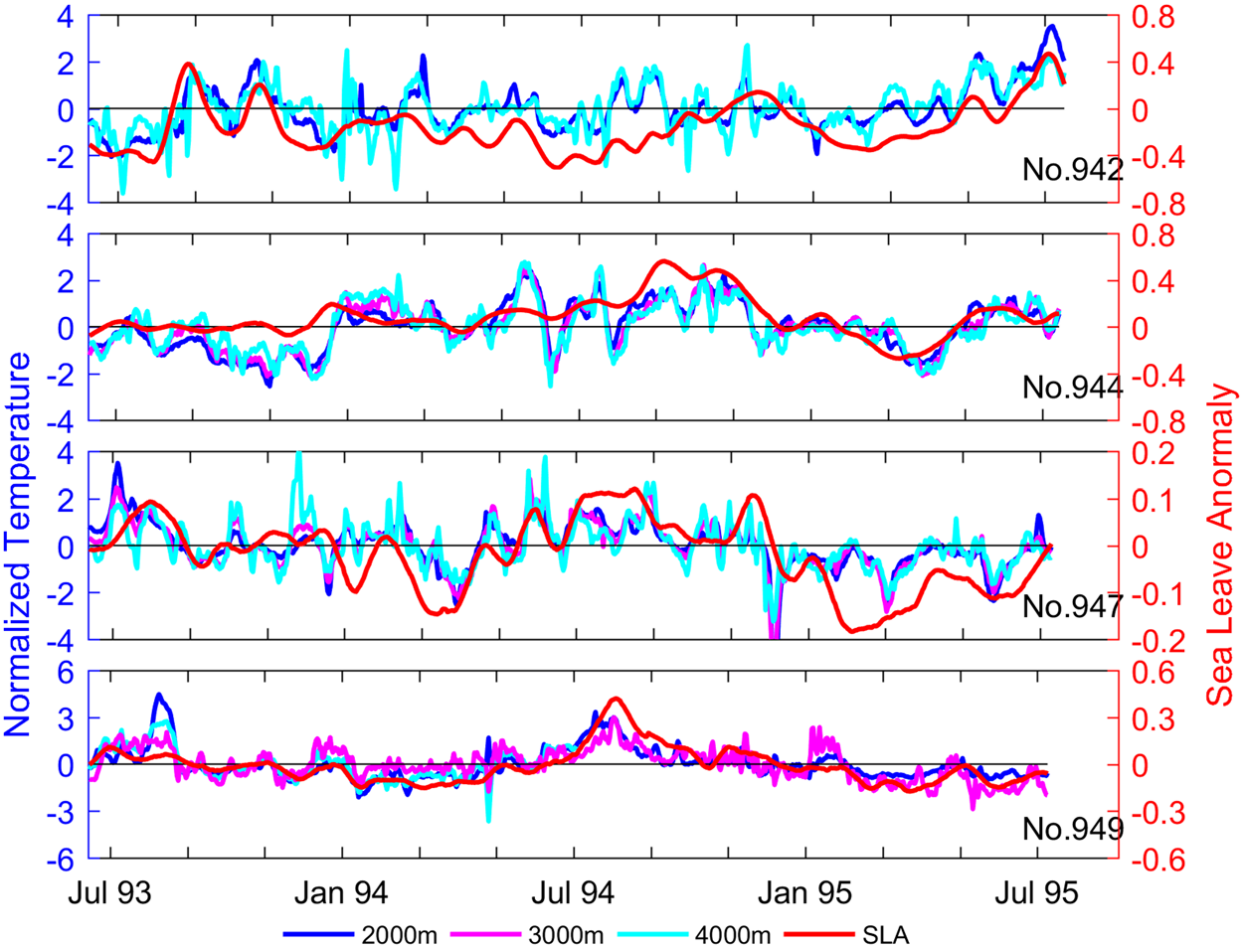
Time series of SLA (red) and NT at 2000 m (blue), 3000 m (cyan) and 4000 m (magenta) from CM Nos. 942, 944, 947, and 949. Figures are plotted using MATLAB R2016a (http://www.mathworks.com/).

The correlation coefficients between SLA and NT at all recorded depths are shown in Table 2. Among the CMs in the abyssal plain (CMs Nos. 942–948), the maximum correlation coefficient is 0.70 (significant above the 95% confidence level) at 2000 m of CMs No. 942 and No. 943. The minimum correlation coefficient is 0.46. In the trench (CM No. 949), the correlation coefficient is up to 0.80 at 3000 m, but the recorded time series covered only one year. On the continental slope (CM No. 950), the topography may be the reason of low correlation coefficient. Furthermore, the correlation coefficient decreased with depth in the range of 2000–4000 m. For example, the correlation coefficient at CM No. 943 decreased from 0.7 at 2000-m depth to 0.6 at 3000 m depth and to 0.46 at 4000-m depth. To examine further the correlation between SLA and NT in the deep ocean, the time series of NT recorded by CMs Nos. 942, 944, 947, and 949 and SLA nearby the CMs are shown in Fig. 4. The correlation coefficients reveal that the trend of SLA and that of NT in the deep ocean were coincident. Take CM No. 942 for instance, the NT increased when SLA began to increase in August 1993, and decreasd when SLA began to decrease in September 1993. Furthermore, the variability of NT at 2000 m was coincident with that at 4000 m. This provides further evidence for the quasi-barotropic structure in the deep ocean described in the last section. In the trench (CM No. 949), the variations of NT and SLA were also coincident as described by high correlation coefficients, although the power spectra of velocity had no obvious peaks.

**Table 2:** Correlation coefficients between SLA and NT at different depths. * means temperature was not recorded by the current meter.

NT Depth		2000 m	3000 m	4000 m
SLA	No. 942	0.7	*	0.46
	No. 943	0.7	0.6	0.46
	No. 944	0.67	0.59	0.53
	No. 945	0.54	*	*
	No. 946	0.63	*	*
	No. 947	0.59	0.57	0.48
	No. 948	*	*	0.46
	No. 949	0.74	0.8	0.66
	No. 950	0.64	0.27	*

**Figure 5:**
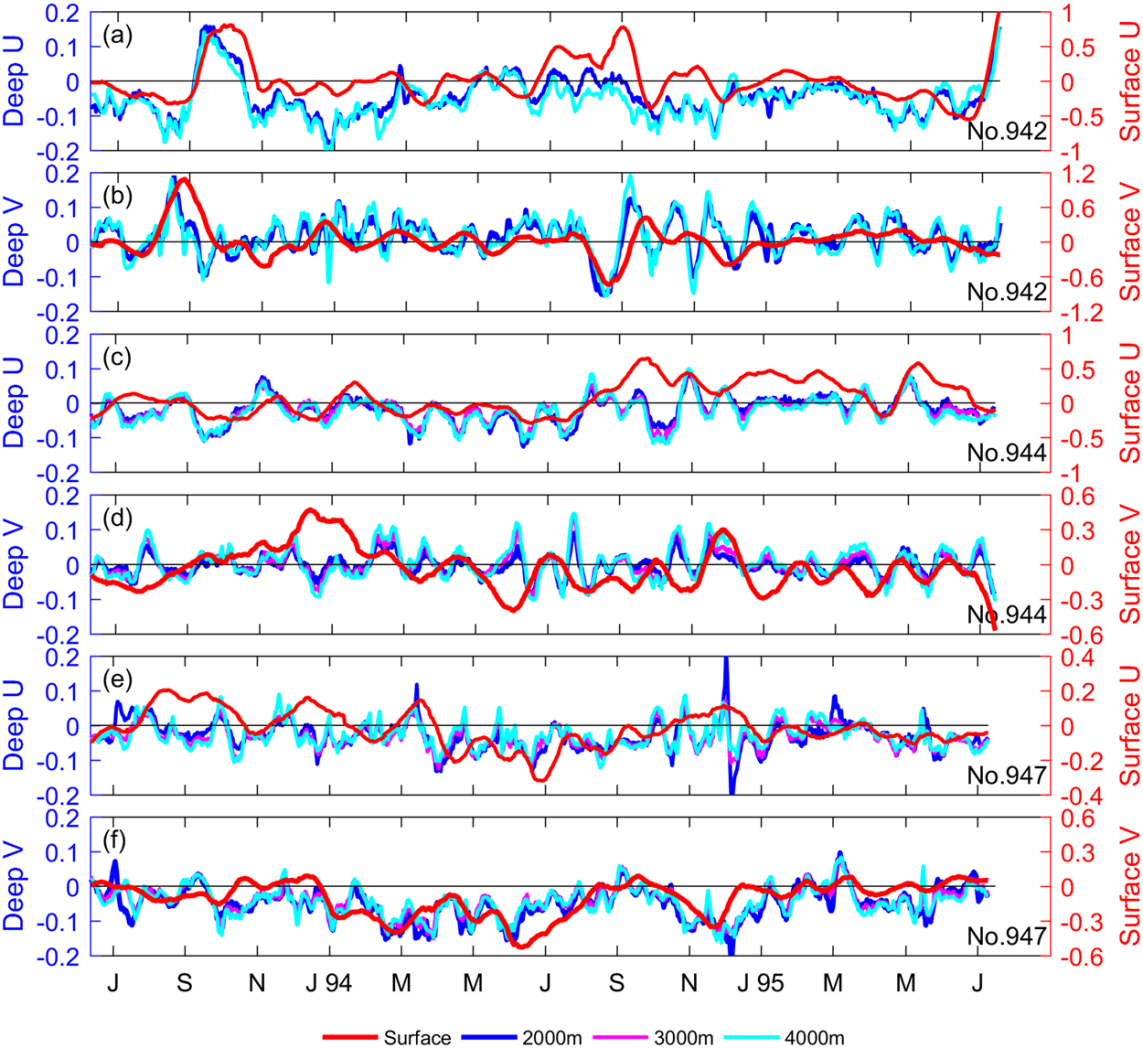
(**a**) Zonal velocities of surface geostrophic current (red) and deep current at 2000 m (blue), 3000 m (cyan) and 4000 m (magenta) at CM No. 942. (**b**) Is the same as (**a**), except for showing the result of meridional velocities. (**c–f**) are the same as (**a,b**), except for showing the results at CM Nos. 947 and 949, respectively. Figures are plotted using MATLAB R2016a (http://www.mathworks.com/).

Next, we compare the geostrophic currents estimated from the SLA near the CMs to the deep currents recorded by the CMs (Fig. 5). The zonal and meridional velocities in both upper and deep oceans exhibited significant low-frequency variability. The variation trend for the deep currents was fairly consistent with that of the geostrophic current, especially when the geostrophic current had significant variation. At CM No. 942, large positive correlation occurred in both zonal and meridional velocities when the geostrophic current increased in August-September 1993 and decreased in August-September 1994. At CMs Nos. 944 and 947, the results also indicated high correlation between surface geostrophic current and deep current; and the maximum correlation coefficient was ~0.57. Furthermore, the zonal and meridional components at different depths of the deep ocean had coincident variability. The barotropic structure below 2000 m was also checked.

### Influence of mesoscale eddies on the deep ocean

**Figure 6:**
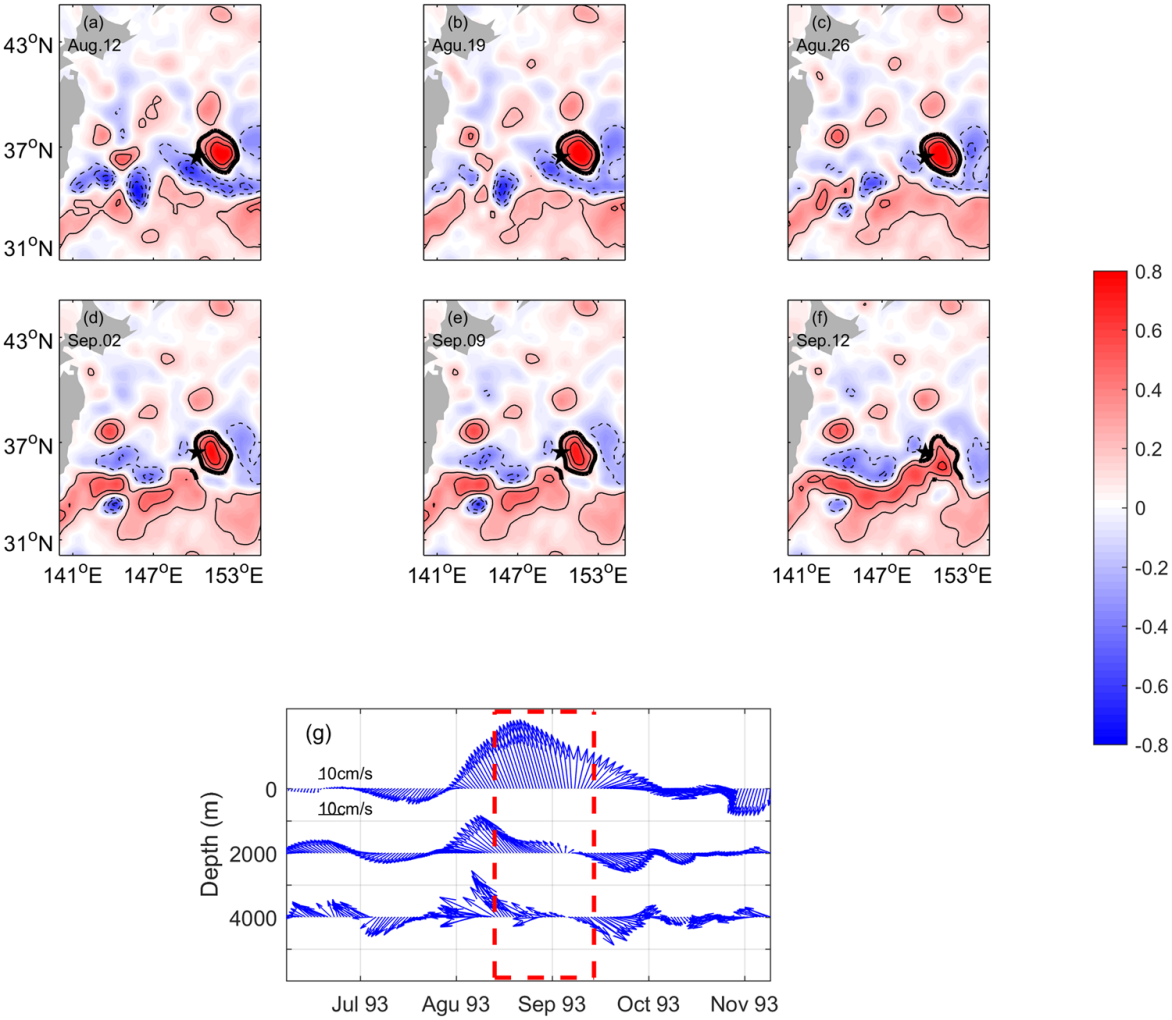
(**a–f**) SLA (m) in the Northwest Pacific from 12 August to 12 September 1993. The black star denotes the location of CM No. 942. The black contours are the SLA, with thick solid, thin solid, and thin dashed lines indicating values equal to, larger than and lower than 0.2 m, respectively. (**g**) Daily SGC time series (0 m) and daily velocities recorded by CM No. 942 (2000, 4000 m) from June to November 1993. Figures are plotted using MATLAB R2016a (http://www.mathworks.com/).

As is known, eddies can be identified from satellite-observed SLA. SLA is positive when an anticyclonic eddy occurs and is negative when a cyclonic eddy is present. To investigate the effect of mesoscale eddies on deep current, the process involving eddies crossing CM No. 942 is illustrated. We compare the SGC estimated from the SLA data near the CM to the currents recorded by the CM. Figure 6 shows the satellite-observed SLA contours and the time series of velocity vector from the upper ocean to the deep ocean when an anticyclonic eddy passed through CM No. 942. An anticyclonic eddy was identified by the SLA center with values greater than 0.2 m. The eddy approached CM No. 942 on August 12, 1993, and then propagated westward, until September 12, 1993 when the anticyclonic eddy vanished. This process is shown in Fig. 6a–f. The sharply increased SLA can also be revealed in the Figs 4 and 5 from August 12, 1993 to September 12, 1993. Note that the southwestern margin of the eddy, rather than the center of the eddy, directly influenced the CM. Therefore, the direction of SGC was northwestward, and the velocity sharply increased when the anticyclonic eddy approached CM No. 942 (Fig. 6g). At depth, the currents at 2000 and 4000 m also exhibited increased amplitude and directed toward the northwest direction. When the eddy moved to the west and departed, the direction of SGC gradually shifted from northwestward to northeastward, and the current speed decreased. It is notable that the current velocity at depth also exhibited distinct variations in both direction and amplitude. The current at 2000-m depth showed a shift from northwestward to northeastward. That directional shift at 4000 m depth as the anticyclonic eddy died off was not obvious as the shift at 2000 m, but it suddenly shifted to southwestward. However, the maximum velocities at both 2000- and 4000-m depths were ~10 cm/s when no eddy was present, and were then evidently enhanced with the maximum velocity reaching ~15 cm/s during the eddy passage.

**Figure 7:**
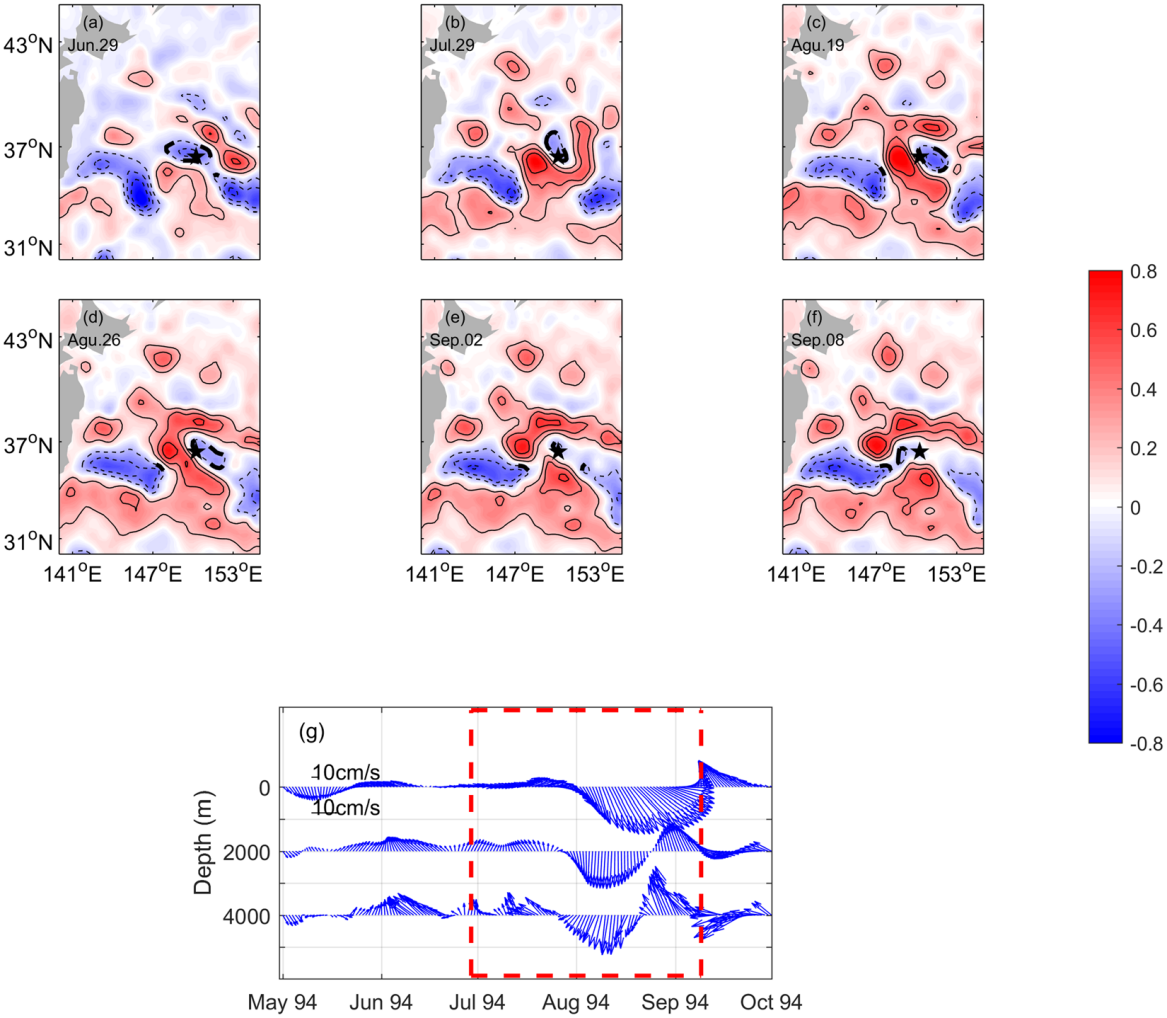
(**a–f**) Similar to Fig. 6 except from 29 June to 8 September 1994. Thick dashed, thin dashed, and thin solid lines indicating values equal to, lower than and larger than −0.2 m, respectively. (**g**) is similar to Fig. 6g, except from May to October 1994. Figures are plotted using MATLAB R2016a (http://www.mathworks.com/).

The other example is when a cyclonic eddy passed through CM No. 942 (Fig. 7). The mooring was located southeast of the cyclonic eddy on June 29, 1994, and the current direction at the surface was east-northeastward. As the eddy moved toward southeast, the mooring was at the west edge of the cyclonic eddy on July 29, 1994. Then, the amplitude of surface current increased as the eddy strengthened. After one month, the cyclonic eddy weakened and died on September 8, 1994. The surface current decreased correspondingly. In the deep ocean, a similar situation was observed for the current velocities at both 2000- and 4000-m depths. When the west margin of the eddy approached CM No. 942 and the eddy enhanced, the amplitude of currents at both 2000- and 4000-m depths increased to ~15 cm s^−1^ and the direction shifted from northeastward to southwestward. The development of the cyclonic eddy can also be seen in Fig. 4. The SLA was negative and absolute value began greater than 0.2 m since June 1994. That means the cyclonic eddy passed through the mooring site.

Whenever an anticyclonic or cyclonic eddy in the upper ocean passed through the mooring site, the deep current data showed that direction shifted and amplitude increased. High consistency between the upper- and deep-current velocities indicates that eddies’ impacts extended beyond the upper ocean to deep ocean. This result confirms the high correlation between SLA and NT mentioned in the previous section.

## Summary and Discussion

In summary, the intraseasonal oscillation of deep currents in the Kuroshio Extension region was investigated using current meter mooring data. Furthermore, the responses in the deep ocean to mesoscale eddies in the upper ocean were investigated by using the satellite observational data. The results of our power spectral analysis, applied to the uninterrupted current meter mooring data of more than two years, revealed 23–38 day peak in both zonal and meridional velocities, and 38–99 day peak in temperature. It means that there existed the intraseasonal (20–100 days) oscillation of deep currents in the Kuroshio Extension region. In addition, the SLA and NT in the deep ocean had high correlation (~0.7). Whether SLA was positive or negative, the NT had the same variation as the SLA, namely, the NT increased (decreased) when the SLA increased (decreased). At the same time, the variability of deep currents was consistent with that of the surface geostrophic current. The locations of the CMs were in the Kuroshio Extension region, where mesoscale eddy often occurs. These mesoscale eddies affect circulations and temperature in the upper ocean. To investigate the effects of the mesoscale eddies on the deep ocean, the process involving eddies crossing the CM was illustrated. During the eddy passed, the velocity of deep-ocean was evidently enhanced. The amplitude became approximately twice the value without an eddy, and the direction showed a strong positive correlation with the surface velocity. That implied the intraseasonal oscillation of mesoscale eddies in the upper layer of the Kuroshio Extension region may have contributed to the intraseasonal oscillation of current and temperature in the deep ocean.

It is worth noting that the mechanisms of the intraseasonal oscillation of deep currents are complicated and remain poorly understood. According to previous studies^13,16^, the deep and surface currents flowed in opposite directions and were highly anti-correlated during eddy events in the South China Sea and near the East Pacific Rise. The explanation for that was the amplitude of the first baroclinic mode rapidly increased when an eddy was generated, and the kinetic energy in the first baroclinic mode became much larger than that in the other modes. Moreover, the deep-penetrating and vertical-tilting eddy structure was found from the observed 3D eddy structure in the South China Sea^31^. That provided the other good explanation for the deep current was intensified and flowed in opposite directions against the surface current. Although the 3D structure is not able to detect from current mooring data and model data, the correlation coefficient between the deep velocity at the mooring site and surface geostrophic velocity at peripheries is calculated. The results revealed that the maximum correlation coefficient was occurred between deep layer at mooring site and surface in the southwest one (Supplementary Table 1). The distance is about 37–39 km, which agrees well with eddies’ propagation distance if the lead time (around 20 days) multiplied by eddies’ propagation speed of 0.02 m/s^32^. But the clear mean structure of eddy needs more observations to get. On the other hand, topographic waves were the other explanation for strong subinertial oscillations generated near the ocean bottom^12^. The other notable point is that the temperature and velocity at deep layer show more high-frequency oscillations than the surface variables. The low-resolution is the directly reason why the altimeter data cannot capture these high-frequency signals. Besides and more probably, subthermocline or deep eddies were observed in Kuroshio Extension region. That may play an important role in modify the deep water properties and be associated with the high-frequency signal in nature subthermocline^33,34^. Notably, high consistency from 2000- to 4000- depth was observed in this study. It means that vertical shear can be neglected and the structure can be considered as quasi-barotropic below the depth of 2000 m. That is consistent with the previous result which given the deep stratification in the Northwestern Pacific is weak. Co-occurrence of passing eddy at the surface and strong velocity at depth indicates that eddy influence extends from the sea surface down to the deep ocean. As previous research, bottom pressure variability on intraseasonal time scales was driven by the atmosphere and ocean instability^23,24^. Therefore, the pressure in the ocean was studied and gradient of pressure is defined as

$$\{\begin{array}{c}\frac{\partial P}{\partial z}=-\,\rho g\\ P{|}_{z=0}={\rho }_{0}g{z}_{0}\end{array}$$

where g is the local gravitational acceleration, ${\rm{\rho }}$ and ${\rho }_{0}$ is water density in depth of z and z = 0 respectively. Therefore, the pressure in depth of z can be given as

1 $${\rm{P}}={\rho }_{0}g{z}_{0}+{\int }_{z}^{0}\rho gdz$$

**Figure 8:**
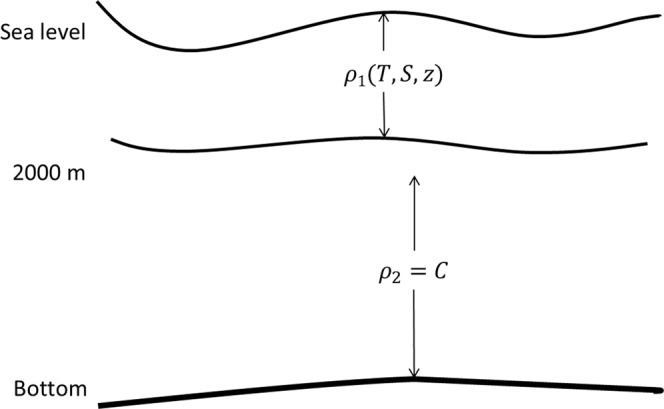
The diagram of vertical density structure.

If the structure can be considered as quasi-barotropic below the depth of 2000 m, Eq. (1) can be re-arranged as

2 $${\rm{P}}={\rho }_{0}g{z}_{0}+{\int }_{2000}^{0}{\rho }_{1}gdz+{\int }_{z}^{2000}{\rho }_{2}gdz$$

where ${\rho }_{1}\,$and ${\rho }_{2}$ is water density above and below 2000 m respectively. ${\rho }_{1}\,$is estimated by temperature, salinity and depth, and ${\rho }_{2}$ is constant and depth-independent. The right hand of Eq. (2) denote the variation of pressure influenced by sea surface fluctuated, barotropic and baroclinic combined in the upper 2000 m, and barotrpic adjusted below 2000 m respectively. Figure 8 shows the diagram of ocean vertical density structure. When the sea surface fluctuated, the deep currents below 2000 m changed due to quasi-barotropic structure. In the upper 2000 m, currents were caused by combined variability from baroclinic and barotropic. But in Kuroshio Extension region, the barotropic variability contributed an important fraction to the current.

Considering the ubiquitousness of eddies in the world oceans, we expect intraseasonal oscillation of deep current and characteristics of mesoscale eddies dominate should similarly be present at other parts of the global ocean. Besides the upper ocean influenced on deep-ocean, other dynamic processes also affected the deep current. The topography waves, ocean mixing, and vertical propagation need to be identified in the future.

## Methods

### Current meter data

The deep current data used in this study were obtained from the WOCE current meter dataset (https://www.nodc.noaa.gov/woce). The observations from WOCE PCM-6 moored current meter for the period from June 1993 to July 1995 are used here. The positions and recorded length of the CMs are listed in Table 1. According the introduction of the dataset, each set of WOCE data was controlled by the Data Assembly Centre (DAC), which has fully described the quality control procedures appropriate to the specific observations. The CM data were examined by the DAC for spikiness and instrument performance. The CMs used in PCM-6 were Neil Brown-EG&G ACM-II acoustic current meters. The accuracy of the ACM-II is ±1 cm s^−1^ or 5% (whichever is greater) for individual velocity samples^9^. For the temperature data, although the recorded accuracy is 0.1 °C, the two significant digit was used as the report form Hallock and Teague^9^. In addition, the conductivity-temperature-depth (CTD) data during the deployment and recovery cruises were used to provide a constant correction to calibrate the bias errors, which may be caused by CM internal register malfunction bits^35^. Therefore, the velocity and temperature recorded by the CMs are of high quality.

### Satellite data

To investigate mesoscale eddies and analyze their surface features, the gridded SLA and surface geostrophic current (SGC) velocity data are used. The multimission altimeter products were produced by Ssalto/Duacs and distributed by the Copernicus Marine and Environment services (CMEMS; http://marine.copernicus.eu/). It merged data from all altimeter missions: Jason-3, Sentinel-3A, HY-2A, and so on. The SLA and SGC data are interpolated onto a global grid of 1/4° × 1/4° resolution with temporal resolution of one day for the entire sampling period of PCM-6 CM observations.

### Data processing and calculations

The original CM data were recorded every 30 minutes from June 8, 1993 to June 29, 1995. As we are interested in the variability on intraseasonal time scales, daily averages are calculated for each CM record to remove high-frequency fluctuations such as diurnal tides, semidiurnal tides and near-inertial currents.

Power spectral analysis is computed by using the relationship that Fourier transformation for the autocorrelation coefficient is power spectral density^36^.

Because the magnitude of temperature anomalies varies with depth, we use NT in the deep ocean to avoid this change. NT is obtained by transforming the mean value and standard deviation of time series to 0 and 1. The method of normalization is achieved by using the statistics function of the Matlab software.

According to a previous study^13^, the temperature in the deep ocean increases (decreases) when the eddy passes through the site, and the SLA is positive (negative) because of vertical pumping rather than the vertical motion of the equipment. To document this, we checked the pressure data recorded by the CMs, which characterize the depth of the equipment. The maximum absolute pressure was no greater than 50 m at CM No. 942 (Supplementary Fig. 2). That means the vertical motion of the equipment was negligible for the temperature variability in the deep ocean.

## Supplementary information

supplementary information
