# Supplementary material for: Intraseasonal oscillation of deep currents influenced by mesoscale eddies in the Kuroshio Extension Region
Source: Sci Rep. 2019 Mar 11;9:4147. doi: 10.1038/s41598-019-39567-7 (PMC6411961; doi:10.1038/s41598-019-39567-7)
Supplement: Supplementary file 1 — supplementary information [file 41598_2019_39567_MOESM1_ESM.docx]

**Intraseasonal oscillation of deep currents influenced by mesoscale eddies in the Kuroshio Extension Region**

**Yansong Liu^1,2^, Fei Yu^1,2,3*^, Feng Nan^1^, Wenzheng Zhou^1,2^**

^1^ Institute of Oceanology, Chinese Academy of Sciences, Qingdao, China

^2^ College of Earth Science, University of Chinese Academy of Sciences, Beijing, China

^3^ Qingdao National Laboratory for Marine Science and Technology, Qingdao, China

* Correspondence and requests for materials should be addressed to F.Y. (yuf@qdio.ac.cn)

For other CMs, both zonal and meridional velocity power spectra indicate 25-33 day peaks and temperature data reveal peaks at around 50 days. (Supplementary Fig. 2).


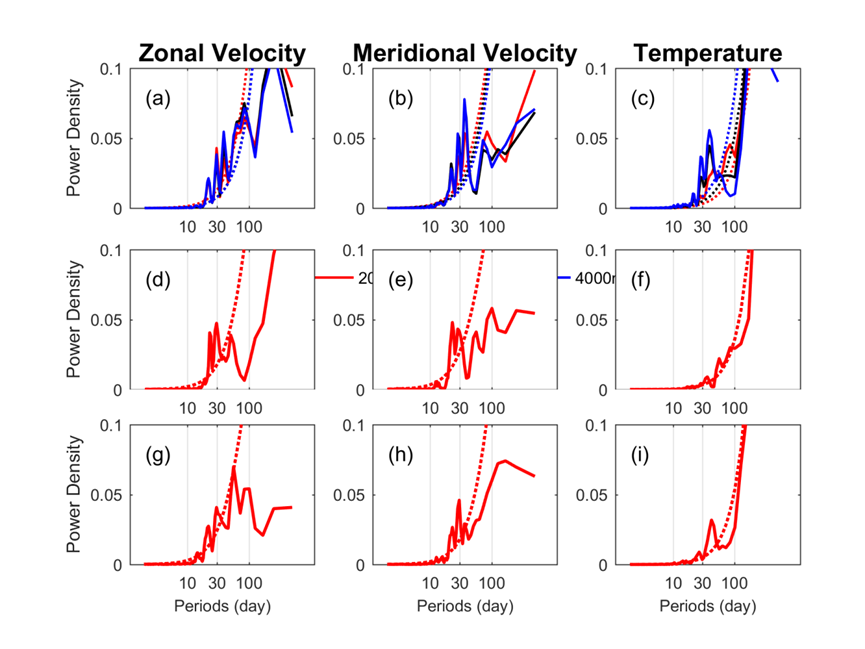


Supplementary Fig. 1 Power spectra density (solid line) and 95% confidence level (dashed line) of zonal velocity (left column), meridional velocity (middle column) and temperature (right column) from CMs No. 943 (a-c), 945 (d-f) and 946 (g-i).

To document weather the temperature in the deep ocean increases (decreases) due to the vertical motion of the equipment, we checked the pressure data recorded by the CMs, which characterize the depth of the equipment. The maximum absolute pressure anomaly was no greater than 50 m at CM No. 942 (Supplementary Fig. 1).

Supplementary Fig. 2 Time series of pressure anomaly and NT at CM No. 942. Blue and red line denotes pressure anomaly and NT, respectively.

We also calculate the correlation between deep velocity at the mooring site and surface geostrophic velocity at peripheries. The results revealed that the maximum correlation coefficient was occurred between deep layer at mooring site and surface in the southwest one (Supplementary Table 1). The distance is about 37-39 km, which agrees well with eddies’ propagation distance if the lead time (15-20 days) multiplied by eddies’ propagation speed of 0.02 m/s (Chelton et al., 2007).

Supplementary Table 1. The correlation coefficient between the deep layer at the mooring site and surface at peripheries. The table in the top row denotes the position in the deep layer which was used to calculate the correlation coefficient with the surface values at mooring site. The tables in the below row are the correlation coefficient between SLA and deep NT, zonal velocity of SGC and deep current and meridional velocity of SGC and deep current, respectively.

| Northwest | North | Northeast |
| --- | --- | --- |
| West | Mooring site | East |
| Southwest | South | Southeast |

| SLA & NT | | |
| --- | --- | --- |
| 0.61 | 0.61 | 0.55 |
| 0.74 | 0.7 | 0.62 |
| 0.78 | 0.76 | 0.68 |

| SGC-V & Deep V | | |
| --- | --- | --- |
| 0.27 | 0.35 | 0.39 |
| 0.41 | 0.43 | 0.43 |
| 0.45 | 0.47 | 0.45 |

| SGC-U & Deep U | | |
| --- | --- | --- |
| 0.59 | 0.49 | 0.37 |
| 0.71 | 0.65 | 0.55 |
| 0.7 | 0.69 | 0.66 |
